# Controlling false discovery rates in factorial experiments with between-subjects and within-subjects tests

**DOI:** 10.1186/1756-0500-6-204

**Published:** 2013-05-21

**Authors:** Eric D Schoen, Carina M Rubingh, Suzan Wopereis, Marjan van Erk

**Affiliations:** 1 TNO Earth, Environmental and Life Sciences, PO Box 360, 3700 AJ Zeist, Netherlands; 2 Department of Environment, Technology and Technology Management, University of Antwerp, Prinsstraat 13, 2000 Antwerp, Belgium

**Keywords:** Analysis of variance, Between-subjects effects, Factorial experiment, False discovery rate, Within-subjects effects

## Abstract

**Background:**

The False Discovery Rate (FDR) controls the expected number of false positives among the positive test results. It is not straightforward how to conduct a FDR controlling procedure in experiments with a factorial structure, while at the same time there are between-subjects and within-subjects factors. This is because there are *P*-values for different tests in one and the same response along with *P*-values for the same test and different responses.

**Findings:**

We propose a procedure resulting in a single *P*-value per response, calculated over the tests of all the factorial effects. FDR control can then be based on the set of single *P*-values.

**Conclusions:**

The proposed procedure is very easy to apply and is recommended for all designs with factors applied at different levels of the randomization, such as cross-over designs with added between-subjects factors.

**Trial registration:**

NCT00959790

## Findings

The control of false positive test results has enjoined considerable attention in the statistical literature. For an overview of methods in case there are many comparisons among treatments, we refer to [1]. More recently, Benjamini and Hochberg [2] and Storey and Tibshirani [3] proposed procedures that control the False discovery Rate (FDR). This is the expected fraction of false positive results among all positive results. The procedures are particularly suited for the analysis of multiple response variables. However, they do not address explicitly the case that there are several tests for one and the same response variable, let alone the presence of several sources of random variation that are to be used for the tests. The purpose of the present communication is to develop an explicit procedure for this case.

### Motivating example

Recently, a study involving human volunteers was conducted at TNO (Zeist, the Netherlands). The study has been carried out in compliance with the Helsinki Declaration, it has been approved by METOPP, Tilburg, the Netherlands, which is an independent centralized ethics committee, and it has been registered at Clinicaltrials.gov, number NCT00959790. The subjects were healthy, non-smoking males aged 18–45 years. All study participants signed an informed consent form. Subjects received financial compensation for their participation.

In the study, subjects from two body mass index (BMI) categories were recruited. Here, we work with the results of 14 obese subjects and 14 lean subjects. The BMI categories define a between-subjects factor at two levels.

Each of the subjects participated in the study during two consecutive periods. Two different diets were given to each subject, one in each period, according to a cross-over design. The diet defines a within-subjects factor, and its effect is to be evaluated against a random error within subjects.

On the last day of each period, subjects completed a physical exercise test. At three time points, blood samples were taken. This defines a within-period factor ‘time’, which is a repeated measurement factor.

Levels of 21 oxylipids were determined in the blood samples; the 168 samples were processed in a completely randomized order.

### Statistical model

The data were studied using the following statistical model.

(1) $$y_{\text{pqr}}=\mu_{\text{pqr}}+e_{2p}+e_{1\text{pq}}+e_{0\text{pqr}},$$

with

(2) $$\begin{array}{rll} \mu_{\text{pqr}}=\mu_{0} & +\beta B_{p}+\delta D_{\text{pq}}+\gamma B_{p}D_{\text{pq}}+\tau_{r}T_{r}+\eta_{r}T_{r}B_{p}\; & \;\\ & +\theta_{r}T_{r}D_{\text{pq}}+\kappa_{r}T_{r}B_{p}D_{\text{pq}}.\; \end{array}$$

In formula (1), *y*_*pqr*_ is the level of an oxylipid from subject *p* (*p*=1…28), period *q* (*q*=1,2) and time *r* (*r*=0,1,2). The measurement is the sum of an expected value modeled with *μ*_*pqr*_ and random contributions modeled with the terms *e*_2*p*_, *e*_1*pq*_, and *e*_0*pqr*_.

The expected value of the measurement *y*_*ijk*_ is detailed in formula (2). We make a distinction between parameters, which are to be estimated from the data, and experimental variables, which indicate the BMI group, the diet, and the time point relevant to the observation. There are 11 parameters, given in Greek alphabet, and four experimental variables, given in Latin alphabet. First, the average difference between the lean and obese groups is modeled with parameter *β* and experimental variable *B*_*p*_. This variable takes the value 1 if subject *p* is obese and 0 otherwise. The parameter *β* thus models the increase in oxylipid level for an obese subject relative to a lean subject.

The average difference between diet 1 and diet 2 is modeled with parameter *δ* and experimental variable *D*_*pq*_. This variable takes the value 1 if subject *p* is given diet 2 and 0 otherwise. The parameter *δ* thus models the increase in oxylipid level for diet 2 relative to diet 1.

Next, the parameter *γ* models the interaction between diet and BMI group. If *γ*=0, the difference between the diets does not depend on the BMI group. If *γ*≠0, the difference between the diets depends on the BMI group.

The parameters that model the average change over time are *τ*_1_ and *τ*_2_, respectively (*τ*_0_ is taken to be zero). The corresponding experimental variables are *T*_1_ and *T*_2_. The first of these takes the value of 1 at time point 1 and 0 otherwise; the second experimental variable takes the value of 1 at time point 2 and 0 otherwise. So the time changes are modeled relative to time point 0.

Further, the parameters *η*_*r*_, *θ*_*r*_ and *κ*_*r*_ model the interaction between BMI group and time, the interaction between diet and time and the three-factor interaction between BMI group, diet and time, respectively.

The three random terms in formula (1) model the random error between subjects, the random error within subjects and the random error within periods, respectively. We assume that the three random terms are independent of each other and normally distributed with variances $\sigma_{2}^{2}$, $\sigma_{1}^{2}$ and $\sigma_{0}^{2}$, respectively.

The subjects can be considered as random samples from two specific populations. Therefore, the 28 *e*_2*p*_ are independent and we can validly carry out an *F* test to assess the difference in BMI level between the two populations.

Further, the subjects were randomly allocated to a treatment order. Therefore, the 28 differences *e*_1*p*1_−*e*_1*p*2_ are independent and we can validly carry out *F* test to assess the effect of diet and its interaction with BMI group.

There could not be a random allocation of the time points to the blood samples. For this reason, the correlations between *e*_*pq*0_ and *e*_*pq*1_, between *e*_*pq*0_ and *e*_*pq*2_, and between *e*_*pq*1_ and *e*_*pq*2_ might not be equal. This would invalidate the analysis of variance *F* tests for the main effect of time and the interactions involving time. Fortunately, the problem posed by unequal correlations can be solved by applying a correction factor to the degrees of freedom for the *F*-tests due to Greenhouse and Geisser [4].

Sometimes, other assumptions on the random terms are reasonable, which may lead to other denominators of the *F* tests being appropriate. We refer to [5] for an extensive discussion of this issue.

### Analysis of variance

An analysis of variance for one of the oxylipids, namely arachidonic acid, is given in Table 1.

**Table 1:** Analysis of variance for arachidonic acid

**Error stratum**	**Source of variation**	**df**	**MS**	***F***_***ij***_	***P***_***ij***_
Between subjects	BMI	1	5.4860	9.98	0.004
	error	26	0.5501		
Wi thin subjects	diet	1	0.0091	0.05	0.8277
	BMI × diet	1	0.6465	3.43	0.0756
	error	26	0.1887		
Within periods	time	2	4.7359	80.77	<0.001
	BMI × time	2	0.0508	0.88	0.3999
	diet × time	2	0.0448	0.76	0.4453
	BMI × diet × time	2	0.1538	2.62	0.08963
	error	104	0.0586		

NOTE: Greenhouse-Geisser ***ε*** = 0.8103.

The first two columns of the table lists the three error strata and the 10 sources of variation present in the data. An error stratum collects all effects that are tested against the same variance; see [6] for a formal definition of a stratum.

All the effects that are measured by contrasting subjects are in the between-subjects stratum. The difference between the groups, which constitutes the BMI main effect modeled with *β* in formula (2), is tested against the random error between subjects.

Each of the two diets was given to each of the subjects. For this reason, the main effect of diet (modeled with *δ* in formula (2)) and the interaction between BMI group and diet (modeled with *γ*) are tested against the random error within subjects.

Finally, the three time points at which blood samples were taken define a third factor, time, whose main effect (modeled with *τ*_1_ and *τ*_2_) is to be tested against a random error within periods. The interactions between BMI category and time (modeled with *η*_1_ and *η*_2_), and between diet and time (modeled with *θ*_1_ and *θ*_2_) are also tested against this random error. The same is the case for the three-factor interaction (*κ*_1_ and *κ*_2_). All these effects are in the within-periods stratum.

Further columns in the table give the degrees of freedom (df) for each source of variation, the corresponding mean square (MS), the value of the individual *F*-ratio (*F*_*ij*_), and the *P*-value (*P*_*ij*_). The index *i* points to the error stratum, while the index *j* points to the *F*-test within a stratum.

The four *F*-tests in the within periods stratum were carried out using the Greenhouse-Geisser *ε* as a correction factor to the degrees of freedom. The calculation of this factor is implemented in most major statistical packages. Here, *ε*=0.8103. Accordingly, the degrees of freedom needed for the calculation of the *P*-values for time and its interactions with the other two factors were 0.8103×2=1.6206 for the numerator and 0.8103×104=84.2712 for the denominator.

Under an individual false positive error rate of 0.05, the outcome for the main effects of BMI and time are highly significant. There is no evidence that the main effect of diet or any interaction effect is statistically significant.

### FDR in factorial experiments with a single stratum

A factorial structure of the study design permits the evaluation of main effects and interactions. For two factors and *m* response variables there are thus 3 *m* tests to carry out. The tests for main effects might not be needed once the interaction is declared statistically significant. This is an important notion, because the total number of the tests is a parameter for the FDR procedure. One could start with a procedure for the *m* tests on active interactions only. In a second step, the variables with significant interactions, *s*_1_, say, are removed from further consideration, and we are left with *m*−*s*_1_ variables not having a proven interaction among the factors. We could then consider applying the FDR procedure on 2(*m*−*s*_1_) main effect tests. However, it is unclear what the performance criteria of the joint first and second step are.

To circumvent the above problem, we propose to replace the three tests with one omnibus *F*-test to see whether the treatments differ. So we initially forget about the factorial structure of the treatments and just check whether there are differences between the treatment groups. For the responses where this is indeed the case, we suggest a follow up that does use the factorial structure, and assess the main effects and interactions using the corresponding *P*-values.

The proposed replacement of individual statistical tests can be carried out easily if all the comparisons between the experimental groups are tested against one and the same error. This is the case if there is just one error stratum, but also if there are several strata while the effect tests involve only one stratum. However, the proposed replacement is not straightforward to apply when effects are tested in several strata. For example, in the motivating study, the error used to test the contrast between lean and obese is different from the error used to test the contrast between the diets. This issue is discussed next.

### FDR in factorial experiments with several strata

We propose calculating a combined *P*-value over all the *F* tests of a response variable as follows: 

1. Denote the number of error strata with *E*, and let *i*=1,…,*E* index the error strata.

2. Let *t*_*i*_ be the number of *F*-tests carried out in stratum *i*. Let *F*_*ij*_ denote the *F*-value of *F*-test *j* in stratum *i*, let *d*_*i*_ denote the degrees of freedom of the denominator, and let *n*_*ij*_ denote the degrees of freedom of the numerator. Calculate $F_{i}=(\sum_{1}^{t_{i}}n_{\text{ij}}F_{\text{ij}})/\sum_{1}^{t_{i}}n_{\text{ij}}$. Under the null hypothesis, this is an *F* statistic with $n_{i}=\sum n_{\text{ij}}$ degrees of freedom for the numerator and *d*_*i*_ degrees of freedom for the denominator.

3. Suppose that the combined *F*-test in stratum *i* has an associated *P*-value of *P*_*i*_. So $P({\underline{F}}_{\left[n_{i},d_{i}\right]}>F_{i}|H_{0})=P_{i}$. Under the null hypothesis, *P*_*i*_∼*U*(0,1), where *U*(*a*,*b*) denotes a uniform distribution with minimum *a* and maximum *b*.

4. Combine the *P*-values by calculating $T_{E}=-2\ln(\prod_{i=1}^{E}P_{i})$.

5. The overall *P*-value is $P(X_{\left[2E\right]}^{2}>T_{E})$, where $X_{\left[2E\right]}^{2}$ is a random variable following a $\chi_{\left[2E\right]}^{2}$ distribution.

6. Apply an FDR control method to the list of overall *P*-values.

7. For variables selected in step (6), study all *P*_*ij*_ to see which factors or interactions contributed to the significance of *T*_*E*_.

The procedure to combine *P*-values is due to Fisher [7]. See [8] for other options to combine *P*-values. The crucial condition for a correct application of Fisher’s procedure is the independence of the *P*-values. This condition is satisfied if the tests involve different error strata.

Step 6 in our procedure results in a set of variables with an expected fraction of at most *α* of false positive results among all positive results, where *α* is the desired level of protection. So the FDR procedure selects variables that show factorial effects. However, the FDR procedure does not operate on the overall list of decisions based on the individual *P*_*ij*_ studied in Step 7. In this aspect, our procedure is analogous to Fisher’s protected least significance difference procedure [1] in one-way analysis of variance, because, in the latter procedure, differences between treatment groups are tested only if the overall *F*-test is statistically significant.

### Application

We apply the proposed procedure to the arachidonic acid response of the motivating example. In the between-subjects stratum, there is nothing to combine, because there is just a single test carried out in this stratum. Recall that the *P*-value for the main effect of BMI is 0.004.

The two *F*-tests in the within-subjects stratum are combined by adding the mean squares of 0.0091 and 0.6465, dividing by 2, and dividing the result by the error mean square of 0.1887. The *F*-value for this stratum is 1.74, with two degrees of freedom for the numerator and 26 degrees of freedom for the denominator. The *P*-value is 0.20. This *P*-value suggests an absence of treatment effects.

For the within-periods stratum, we multiply the mean squares for time, BMI × time, diet × time, and the three-factor interaction BMI × diets × time with 2, add up and divide by 8. This results in a combined mean square of 1.2463. This mean square is tested against the error mean square, giving an *F*-value of 21.26, based on 8 and 104 degrees of freedom. The degrees of freedom were corrected with the Greenhouse-Geisser *ε* statistic to 6.4824 and 84.2712, respectively. The associated *P*-value is nearly zero. For further processing we replaced this with a value of 10^−16^.

Finally, the three *P*-values are to be combined to one overall value. We take –2 times the natural logarithm, and add up. This gives *X*^2^=87.945. The reference distribution for this statistic is the $\chi_{\left[6\right]}^{2}$ distribution. The statistic has a *P*-value of 8.09×10^−17^.

**Figure 1:**
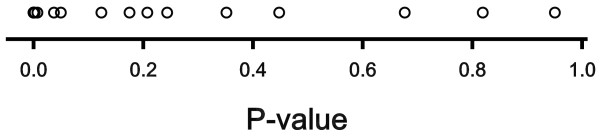
***P*-values for 21 oxylipids.** Each circle represents an overal *P*-value for a particular oxylipid, summarizing the results of 7 statistical tests.

**Figure 2:**
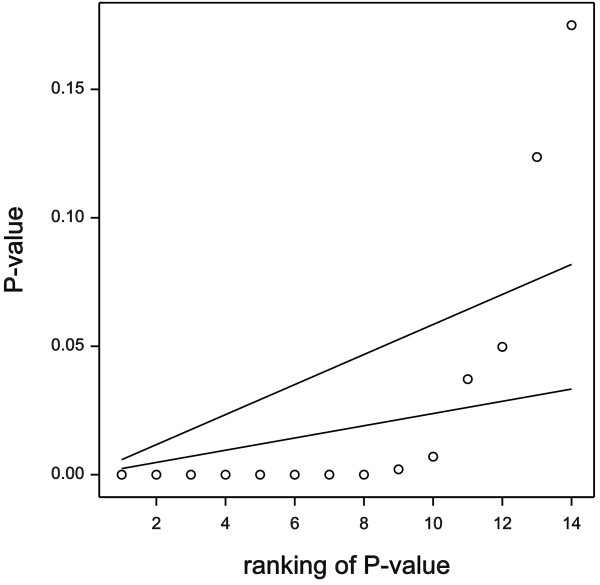
**Rejections for two FDR procedures.**P-values below lower line: rejected by the Benjamini-Hochberg procedure [2]; *P*-values below upper line: rejected by the Storey-Tibshirani procedure [3].

All the overall *P*-values according to the proposed procedure are shown in Figure 1. For 12 of the oxylipids, including arachidonic acid, *P*<0.05. The application of the FDR-controlling procedures of [2] and [3], is visualized in Figure 2. The *P*-values are ordered and plotted against their order number. We restrict attention to the values below 0.2, and we use a boundary value of 0.05 for both procedures. The lower line gives the boundary values for the Benjamini-Hochberg procedure [2]. The largest *P*-value below the line has order number 10. So the procedure reveals that 10 out of 21 oxylipids are affected by the experimental factors. The upper line in Figure 2 bears on the procedure of Storey and Tibshirani [3]. When compared with the Benjamini-Hochberg procedure, two more *P*-values are included in the set with *q*<0.05. Note that the set now includes all oxylipids for which *P*<0.05. This is not generally the case, however.

Some authors would favor error-control methods that are more conservative than FDR. For example, the well-known Bonferroni correction would compare all 21 combined *P*-values with an error rate of 0.05/21. Clearly, the proposed FDR methods are more lenient than the Bonferroni correction in declaring that a variable is significantly affected by the study factors.

We like to point out that both FDR controlling procedures are sensitive to strong negative correlations between the *P*-values; see [9]. For the oxylipids, this is not really an issue because the average pairwise correlation among the oxylipids was +0.1. With three exceptions, all correlations were above –0.3; the smallest exceptional value was –0.5. We therefore think that our application of the FDR control is justified.

As a final issue, we had an equal interest in all the oxylipids and all the model parameters. In case of variables or parameters of primary interest, one option is to include only these variables or parameters. This will make the procedure more powerful, because non-significant values of the *F* statistic that are not of interest will tend to reduce the overall test statistic. Alternatively, there are options to introduce weights to the variables or parameters other than 1 for those of primary interest and 0 for those of secondary interest. However, a discussion of these options is beyond the scope of the present paper.

## Availability of supporting data

The data set supporting the results of this article is included within the article and its additional file called FDR_overall_Pvalue_calculation.xlsx. The additional file shows for each of the 21 oxylipids, first the *F*_*ij*_ values arranged in seven rows and 21 columns. The columns correspond to the oxylipids and the rows correspond to the seven statistical tests for each individual oxylipid. Next, the 21 values for the Greenhouse-Geisser epsilon statistic are given. Then we give the *P*-values for each of the three error strata arranged in three rows and 21 columns. The columns correspond to the oxylipids and the rows correspond to the between-subjects, within-subjects and within-period strata, respectively. Finally, we give the value of the statistic *T*_*E*_, as calculated in step 4 of the proposed procedure, and the corresponding overall *P*-value for the factorial effects of the 21 oxylipids.

## Abbreviations

BMI: Body mass index.

## Competing interests

The authors declare that they have no competing interests.

## Authors’ contributions

EDS formulated the proposed procedure and conducted a detailed analysis of the arachidonic acid response. CR wrote computer code to apply the proposed procedure. SW and MvE designed and conducted the oxylipid study. All authors read and approved the final manuscript.

## References

[B1] Hochberg Y Tamhane A Multiple comparison procedures 1987 New York: Wiley

[B2] Benjamini Y Hochberg Y Controlling the false discovery rate: a practical and powerful approach to multiple testing J R Stat Soc Ser B 1995 57 289 300

[B3] Storey JD Tibshirani R Statistical significance for genomewide studies Proceedings of the National Academy of Sciences USA 2003 100 9440 9445 10.1073/pnas.1530509100 PMC170937 12883005

[B4] Greenhouse SW Geisser S On methods in the analysis of profile data Psychometrika 1959 24 95 112 10.1007/BF02289823

[B5] McLean RA Sanders WL Stroup WW A unified approach to mixed linear models Am Statistician 1991 45 54 64

[B6] Bailey RA Design of comparative experiments 2008 Cambridge: Cambridge University Press

[B7] Fisher RA Statistical methods for research workers 1935 London: Oliver and Boyd

[B8] Fang Y Wit E Tang C, Ling C, Zhou X, Cercone N, Li X Test the overall significance of p-values by using joint tail probability of ordered p-values as test statistic Advanced Data Mining and Applications Volume 5139 of Lecture Notes in Computer Science 2008 Heidelberg: Springer Berlin 435 443

[B9] Benjamini Y Yekutieli D The control of the false discovery rate in multiple testing under dependency Ann Stat 2001 29 1165 1188 10.1214/aos/1013699998

